# IFMA-Net: Interactive Feature Modulated Attention Network for Accurate Identification of Cotton Pests and Diseases from Field Images

**DOI:** 10.3390/s26196026

**Published:** 2026-09-23

**Authors:** Lili Song, Zhenxue Chen, Chengyun Liu, Yixin Guo, Dongke Wang

**Affiliations:** 1 School of Control Science and Engineering, Shandong University, Jinan 250061, China; 2 School of Energy and Control Engineering, Changji University, Changji 831100, China; 3 Xinjiang Research Institute, Shandong University, Changji 831100, China

**Keywords:** cotton pests and diseases identification, agricultural vision sensing, attention mechanism, cross-attention fusion, deep learning

## Abstract

Recognizing cotton pests and diseases from field-captured images remains challenging due to subtle symptoms, complex backgrounds, high intra-class variation, and considerable variation in symptom scale. This study proposes IFMA-Net, a ResNet-50-based network that integrates local feature enhancement, multi-scale contextual modeling, and interactive feature fusion for agricultural vision sensing. The Dynamic Local Enhancement (DLE) module enhances discriminative shallow features, while the Global Multi-Scale Attention and Local Aggregation (G-MSLA) module combines multi-scale local aggregation with global dependency modeling. The Interactive Cross-Attention Fusion (ICAF) module employs two opposing cross-attention branches to exchange information between local and global representations. On a self-constructed eight-class field dataset, IFMA-Net achieved 95.53 ± 0.17% accuracy and 94.81 ± 0.14% macro-F1, exceeding ResNet-50 by 5.52 and 7.32 percentage points. Ablation experiments confirmed the individual contributions of the three modules. On an independent public test set, IFMA-Net achieved 90.21% macro-F1. Perturbation experiments showed higher retention than ResNet-50 under random occlusion but substantial degradation under severe Gaussian noise. These results demonstrate IFMA-Net’s potential for accurate pest and disease identification in practical agricultural sensing scenarios.

## 1. Introduction

Cotton is a major economic crop, and its yield and quality directly affect the supply of raw materials for the textile industry, agricultural profitability, and associated supply chains [1,2]. Compared with conventional manual inspection, vision-based sensing and automated recognition of pests and diseases are more efficient, less costly, and easier to deploy at scale in agricultural fields. Therefore, automated recognition of cotton pests and diseases under natural field conditions is important for enhancing early-warning capabilities, reducing the burden of manual inspection, and supporting stable cotton production and grower income [3,4].

Artificial intelligence has advanced rapidly in agricultural image analysis, and neural networks are now a core technology for visual perception and recognition in smart agriculture. More recently, foundation models have shown promise in agricultural scenarios because of their transferability and generalization ability, although annotation costs and field complexity remain major challenges. For crop pest and disease recognition, recent studies have demonstrated that deep learning can substantially improve classification performance [5,6]. Early deep learning approaches relied primarily on CNN-based architectures such as AlexNet [7], VGG [8,9], ResNet [10], and EResNet [11], often combined with transfer learning to achieve strong performance on benchmark datasets. However, these models remain sensitive to illumination changes, occlusion, background clutter, and scale variation under natural field conditions. Their robustness is particularly limited when images contain complex backgrounds, subtle symptoms, and lesions of different sizes.

To suppress background interference and highlight symptom-related regions in field images, researchers have introduced channel, spatial, and hybrid attention mechanisms [12]. Vision-sensing models enhanced with attention mechanisms have also shown promising results in detecting crop pests and diseases under complex field conditions [13,14]. Modules such as SE, CBAM, non-local blocks, and ASPP have been applied to improve feature selection and contextual modeling [15,16]. Although these methods have enhanced symptom representation, many still rely on global statistics or single-scale features, thereby limiting their ability to capture low-saliency symptoms in complex field scenes. Moreover, many designs emphasize one-way top-down enhancement and do not sufficiently promote bidirectional interaction between shallow detail features and deep semantic features.

To address substantial variation in symptom size in field-acquired images, previous studies have employed multi-branch convolutions, dilated convolutions, and pyramid structures to expand receptive fields and enrich multi-scale representations. FPN and BiFPN variants have proven effective in fusing multi-level semantic features, while Transformer-based self-attention has more recently been introduced to capture global dependencies [17]. Nevertheless, in complex field scenes, shallow and deep features are often misaligned, and static fusion strategies can introduce redundant or conflicting information. In addition, interpretability is essential for evaluating the reliability of agricultural visual recognition models. Visualization methods such as Grad-CAM are widely used to identify discriminative regions and assess whether models focus on symptom-relevant areas [18,19]. However, the relationship between visualization results and model architecture, including feature enhancement, multi-scale modeling, and fusion strategies has received comparatively limited attention. Together, these gaps indicate that adaptive feature modulation and collaborative cross-layer modeling remain underexplored for fine-grained cotton pest and disease recognition.

Cotton field pest and disease images pose visual challenges that differ markedly from those in classification datasets collected under controlled or simple-background conditions [20,21]. Many symptoms appear as small, scattered, low-contrast spots and subtle texture changes that are easily lost during repeated downsampling. Moreover, leaf folding, mutual occlusion, and substantial variation in symptom size disrupt the integrity of local visual patterns, making it difficult for features based solely on fixed receptive fields to reliably represent these patterns [22]. In addition, complex backgrounds containing soil, branches, and other leaves, together with variations in illumination, can generate responses that resemble disease-related textures. Shallow fine-grained details and deep semantic information also differ in both spatial resolution and level of abstraction [23,24]. Therefore, this task requires the simultaneous preservation of local details, modeling of multi-scale context, and effective interaction between shallow and deep representations.

Based on the task characteristics described above, we propose IFMA-Net. Specifically, DLE operates on high-resolution shallow features to preserve the fine-grained textures and boundary information of symptoms. G-MSLA operates on deep features and jointly models symptoms at different scales and the global leaf context through multi-scale atrous convolutions and self-attention. After aligning local and global representations, ICAF promotes complementary interactions between fine-grained details and high-level semantics through two cross-attention branches with opposite information-flow directions. The main contributions of this study are as follows:

(1) We propose IFMA-Net, which jointly models local enhancement, multi-scale contextual awareness, and cross-layer feature fusion for cotton pest and disease recognition.

(2) We design three key modules: DLE for local texture refinement, G-MSLA for multi-scale dependency modeling, and ICAF for interactive feature fusion.

(3) Extensive experiments on an eight-class cotton pest and disease dataset show that the proposed achieves the highest mean classification accuracy and macro-F1 score among the evaluated methods. Ablation studies, robustness evaluations, and visualization analyses further demonstrate its effectiveness and interpretability.

The remainder of this paper is organized as follows. Section 2 describes the dataset construction and the proposed IFMA-Net architecture. Section 3 presents the experimental results and analysis. Section 4 discusses the findings, and Section 5 concludes the paper and outlines directions for future research.

## 2. Materials and Methods

### 2.1. Dataset Construction

While large-scale benchmark datasets such as DLCPD-25 [25] have advanced the development of generalizable crop disease recognition models, they cover multiple crop species with limited class-specific samples for cotton, and cotton-specific field datasets remain scarce. Therefore, this study constructs a dedicated eight-class cotton pest and disease dataset for model development, complemented by an independent publicly available dataset for external evaluation. The following subsections describe the construction of the self-built dataset, the selection of the external test dataset, and the image preprocessing and data augmentation procedures.

#### 2.1.1. Self-Constructed Cotton Pest and Disease Dataset

An eight-class cotton pest and disease dataset comprising 5400 field images was constructed. The dataset included Healthy, Aphids, Armyworm, Bacterial Blight, Fusarium Wilt, Grey Mildew, Leaf Curl, and Leaf Spot. The characteristic field symptoms and commonly associated pests or putative causal agents are summarized below, with detailed symptomatological and taxonomic information provided in Supplementary Table S1 (Supplementary Materials).

Healthy images showed uniformly green leaves without visible chlorosis, necrosis, or deformation. Aphid infestation was characterized by dense insect colonies on the abaxial leaf surfaces, accompanied by leaf curling, honeydew deposition, and sooty mold; these symptoms are commonly associated with *Aphis gossypii* Glover. Armyworm damage was characterized by irregular feeding holes, marginal notches, and skeletonized leaves; such damage may be associated with noctuid larvae, particularly *Helicoverpa armigera* and *Spodoptera litura*, although exact species-level identification could not be determined from the field images alone. Bacterial Blight was characterized by angular, water-soaked lesions along the leaf veins that subsequently darkened and could extend to the stems and bolls; these symptoms are commonly associated with *Xanthomonas citri* pv. *malvacearum*. Fusarium Wilt was characterized by progressive yellowing and wilting, often initially affecting one side of the plant; these symptoms are commonly associated with *Fusarium oxysporum* f. sp. *vasinfectum*. Grey Mildew was characterized by angular, pale-green to yellow lesions and white-to-grey sporulation on the abaxial leaf surface; these symptoms are commonly associated with *Ramulariopsis gossypii*. Leaf Curl was characterized by curling of the leaf lamina, vein thickening, and enations; these symptoms are consistent with cotton leaf curl disease (CLCuD), which is commonly associated with a begomovirus complex, including Cotton leaf curl Multan virus, and transmitted by *Bemisia tabaci*. Leaf Spot was characterized by circular to irregular necrotic lesions; similar symptoms are commonly associated with *Alternaria* spp. and *Cercospora gossypina*.

Images were acquired during the 2024 and 2025 growing seasons in major cotton-producing regions of Xinjiang, China, using Huawei Mate 70 smartphones (Huawei Technologies Co., Ltd., Shenzhen, China) and Canon EOS M50 cameras (Canon Inc., Tokyo, Japan). To capture representative phenotypic and environmental variation, images were collected from multiple viewing angles at camera-to-leaf distances of 15–20 cm under sunny, cloudy, and overcast conditions. Image acquisition was conducted between 10:30 and 13:30 and between 17:00 and 19:00 to reduce overexposure and harsh shadows. Image resolutions ranged from 1000 × 1000 to 4000 × 4000 pixels. The dataset included naturally occurring field variability in illumination, background complexity, partial occlusion, viewing angle, and symptom severity.

Data quality was controlled through a two-stage verification process comprising an initial field assessment followed by expert agronomic review. Blurred, ambiguous, or otherwise low-quality images were excluded. The retained images were annotated according to observable field phenotypes and agronomic expertise, without laboratory-based pathogen confirmation. Collection metadata were preserved to maintain traceability. The samples are shown in Figure 1.

Exact duplicate images were identified and removed using SHA-256 hashing. Near-duplicate images were identified using 64-bit perceptual hashing and subsequently verified manually. Images originating from the same or highly similar field samples were assigned to the same subset to minimize data leakage. Stratified sampling with a fixed random seed was used to maintain comparable class distributions and ensure reproducible partitioning. The dataset was divided into training (3784 images; 70.1%), validation (1169 images; 21.6%), and internal test (447 images; 8.3%) sets. The class-wise distributions are summarized in Table S2 (Supplementary Materials).

#### 2.1.2. Independent Public Dataset for External Evaluation

To evaluate cross-dataset generalization, an independent external test was conducted using the open-source Cotton Crop Diseases dataset (CC BY 4.0 license, Roboflow Universe) [26]. The dataset comprised 5752 images across eight disease categories, with official training/validation/test splits of 4026/863/863 images. To ensure semantic consistency with our self-built dataset, we retained only four overlapping categories: Healthy, Aphids, Armyworm, and Bacterial Blight. Only the official test partition was adopted for evaluation, resulting in 437 external test images. The category distribution of the external test set is summarized in Table S3 (Supplementary Materials). Category name formats were unified without altering biological semantics, and classes without a clear counterpart were excluded.

The training and validation subsets of the external dataset were not used for model training or hyperparameter tuning. For external evaluation, only resizing and normalization were applied, with no random augmentation. To ensure a fair comparison, a four-class training protocol was established using the corresponding classes from the self-built dataset, under which the generalization performance of ResNet-50 (the best-performing baseline) and IFMA-Net was compared. Internal four-class results and external test results were analyzed independently and were not combined with the eight-class main experimental metrics.

#### 2.1.3. Image Preprocessing and Data Augmentation

All images were resized to 224 × 224 pixels and normalized using the ImageNet channel-wise mean and standard deviation. Random data augmentation, including rotation (±15°), brightness adjustment (±20%), horizontal flipping, and contrast adjustment, was applied only to the training set. The validation, internal test, and external test sets underwent only resizing and normalization. All models were trained and evaluated using identical data splits and preprocessing procedures.

### 2.2. IFMA-Net for Cotton Pests and Disease Identification

#### 2.2.1. Overall Architecture

Cotton pest and disease images acquired under natural field conditions commonly exhibit subtle symptoms, cluttered backgrounds, scale variations, and substantial intra-class variability. To address these challenges, IFMA-Net is constructed using ResNet-50 as the backbone and integrates local feature enhancement, multi-scale local–global modeling, and interactive feature fusion. The overall architecture is shown in Figure 2.

Given an input image $x\in R^{B\times 3\times 224\times 224}$, the four residual stages generate hierarchical features $F_{1},\;F_{2},{\;F}_{3}$ and $F_{4},$ with dimensions of B×256×56×56, B×512×28×28, B×1024×14×14, and B×2048×7×7, respectively. The intermediate features $F_{2}$ and $F_{3}$ are used only for backbone propagation. The high-resolution feature $F_{1}$ is processed by the Dynamic Local Enhancement (DLE) module to enhance discriminative texture and boundary responses, yielding $F_{\mathrm{local}}\in R^{B\times 256\times 56\times 56}.$ The deep, semantically rich feature $F_{4}$ is processed by the Global Multi-Scale Attention and Local Aggregation (G-MSLA) module to model multi-scale context and long-range dependencies, yielding $F_{\mathrm{global}}\in R^{B\times 2048\times 7\times 7}$.

In the Interactive Cross-Attention Fusion (ICAF) module, $F_{\mathrm{local}}$ and $F_{\mathrm{global}}$ are first aligned in spatial and channel dimensions and then interactively fused via bidirectional cross-attention, producing the fused feature $F_{\mathrm{ICAF}}$. For classification, $F_{\mathrm{ICAF}}$ is projected to 2048 channels and globally pooled to obtain $u_{l}$, while $F_{\mathrm{global}}$ is directly pooled to obtain $u_{g}$. The two B×2048- dimensional representations are concatenated and fed into a fully connected classification head (4096→1024→8), which preserves complementary local–global information at the image level.

The detailed architectures of the three proposed modules are illustrated in Figure 3 and described in Section 2.2.2, Section 2.2.3 and Section 2.2.4.

#### 2.2.2. Dynamic Local Enhancement (DLE) Module

Discriminative information in cotton disease and pest images typically appears as fine-grained features—color shifts, lesion boundaries, and local textures—that are mainly captured in the shallow layers of convolutional networks, where they are easily overwhelmed by complex backgrounds and unstable feature responses. To enhance salient local cues in these shallow features, we introduced a Dynamic Local Enhancement (DLE) module at the output of the first residual stage of ResNet-50. As illustrated in Figure 3a, DLE sequentially applies sample-adaptive channel-wise affine modulation, spatial attention recalibration, and residual feature projection.

Let $F_{1}\in R^{B\times C\times H\times W}$ denote the output feature of the first residual stage, where *B*, *C*, *H*, and *W* represent batch size, channel count, feature map height, and width, respectively. DLE employs two parallel branches to generate sample-adaptive channel modulation parameters. The offset branch applies global average pooling (GAP) and global max pooling (GMP) to $F_{1}$, concatenates the two descriptors (*B* × 2*C* × 1 × 1), and feeds them into an MLP to produce the channel offset $\beta \in R^{B\times C\times 1\times 1}$. The scaling branch applies global average pooling to $F_{1}$ and generates the channel scaling coefficient $\gamma \in R^{B\times C\times 1\times 1}$ via an MLP and sigmoid activation in Equation (1).

(1) $$\begin{array}{l} \beta ={\mathrm{MLP}}_{\beta }\left(\left[\mathrm{GAP}\left(F_{1}\right);\mathrm{GMP}\left(F_{1}\right)\right]\right)\\ \gamma =\sigma \left({\mathrm{MLP}}_{\gamma }\left(\mathrm{GAP}\left(F_{1}\right)\right)\right) \end{array}$$

where $\mathrm{Concat}\left(\cdot \right)$ denotes channel-wise concatenation, $\sigma \left(\cdot \right)$ denotes the Sigmoid function, and both branches employ a channel compression–expansion ratio r (C → C/r → C). The parameters β and γ are then broadcast along the spatial dimensions to perform channel-wise affine modulation in Equation (2).

(2) $$U=\gamma ⊙F_{1}+\beta$$

where ⊙ denotes element-wise multiplication. Because both modulation parameters are derived from the channel statistics of the current input sample, this operation adaptively recalibrates the scale and shift in each feature channel in a sample-dependent manner.

To preserve discriminative spatial responses, DLE computes channel-wise average and max pooling of the modulated feature U. It then concatenates the two resulting spatial feature maps (*B* × 2 × *H* × *W*) and applies a 7 × 7 convolution (stride 1, padding 3; 2 → 1 channels) followed by a Sigmoid function to generate a spatial attention map $\;M_{s}$, as defined in Equation (3).

(3) $$\begin{array}{l} M_{s}=\sigma \left({\mathrm{Conv}}_{7\times 7}\left[{\mathrm{AvgPool}}_{c}\left(U\right);{\mathrm{MaxPool}}_{c}\left(U\right)\right]\right)\\ U_{s}=M_{s}⊙U \end{array}$$

where ${\mathrm{AvgPool}}_{c}\left(\cdot \right)$ and ${\mathrm{MaxPool}}_{c}\left(\cdot \right)$ denote average and max pooling along the channel dimension, respectively. $M_{s}$ is broadcast along the channel dimension and multiplied with *U* element-wise to yield the recalibrated feature $U_{s}$. Finally, $U_{s}$ undergoes a 1 × 1 convolution, batch normalization, and ReLU activation, and is added to the original input $F_{1}$ via a residual connection, as defined in Equation (4).

(4) $$F_{\mathrm{local}}=F_{1}+\mathrm{ReLU}\left(\mathrm{BN}\left({\mathrm{Conv}}_{1\times 1}\left(U_{s}\right)\right)\right)$$

The output $F_{\mathrm{local}}\in R^{B\times C\times H\times W}$ retains the original Stage 1 representation while incorporating channel-modulated and spatially recalibrated information, and serves as the local feature input to the cross-layer fusion module.

#### 2.2.3. Global Multi-Scale Attention and Local Aggregation (G-MSLA)

Visible symptoms of cotton pests and diseases differ markedly in both morphology and spatial extent. A single convolutional scale is therefore often inadequate to capture localized spots, leaf-margin abnormalities, and broader changes in leaf texture. To aggregate local context across multiple receptive-field scales and to model relationships among spatial locations, a Global Multi-Scale Attention and Local Aggregation (G-MSLA) module was introduced after the fourth residual stage of ResNet-50. As illustrated in Figure 3b, G-MSLA sequentially performed multi-scale feature aggregation, global self-attention modeling, and spatial feature reconstruction.

Let the Stage 4 output $F_{4}\in R^{B\times C\times H\times W}$, where C=2048 and H=W=7. Three parallel 3 × 3 depthwise-separable dilated convolution branches used dilation rates of 1, 3, and 5. Their outputs were concatenated along the channel dimension and then compressed from 6144 to 256 channels by a convolution, as shown in Equation (5).

(5) $$\begin{array}{l} F^{\left(i\right)}={\mathrm{DSConv}}_{3\times 3,d_{i}}\left(F_{4}\right),d_{i}\in \left\{1,3,5\right\}\\ F_{\mathrm{ms}}={\mathrm{Conv}}_{1\times 1}\left(\left[F^{\left(1\right)};F^{\left(2\right)};F^{\left(3\right)}\right]\right) \end{array}$$

where [·,;·] denotes concatenation along the channel dimension. Each branch produces a feature map of B×2048×7×7, whereas the fused feature satisfies $F_{\mathrm{ms}}\in R^{B\times 256\times 7\times 7}$. The dilation rates provide different receptive-field scales without imposing a predetermined correspondence between an individual branch and a specific symptom scale. The spatial dimensions of $F_{\mathrm{ms}}$ are flattened, and the channel dimension is transposed to the last axis to form the Transformer input sequence as shown in Equation (6).

(6) $$X=\mathrm{Transpose}\left(\mathrm{Flatten}\left(F_{\mathrm{ms}}\right)\right)\in {\mathbb{R}}^{B\times N\times C^{\prime \prime }},N=49,C^{\prime \prime =256}$$

G-MSLA employs a Pre-Norm Transformer block, in which X is processed sequentially by layer-normalized multi-head self-attention (MHSA) and a feed-forward network (FFN), with a residual connection after each sublayer, as defined in Equation (7).

(7) $$\begin{array}{l} X_{1}=X+\mathrm{MHSA}(\mathrm{LN}(X))\\ X_{2}=X_{1}+\mathrm{FFN}(\mathrm{LN}(X_{1})) \end{array}$$

MHSA uses an embedding dimension of 256, eight attention heads, and a head dimension of 32. The feed-forward network follows the channel mapping 256→1024→256. No additional positional encoding is used in this module. Because the Stage 4 representation contains only 49 spatial tokens, self-attention models interactions among different spatial regions at a relatively low spatial resolution.

The Transformer output $X_{2}$ is reshaped to B×256×7×7, projected back to 2048 channels by a 1×1 convolution, and batch-normalized. The projected feature is then added to the original $F_{4}$ via a final residual connection, as defined in Equation (8).

(8) $$F_{\mathrm{global}}=F_{4}+\mathrm{BN}\left({\mathrm{Conv}}_{1\times 1}\left(\mathrm{Reshape}(X_{2})\right)\right)$$

The output $F_{\mathrm{global}}\in {\mathbb{R}}^{B\times 256\times 7\times 7}$ retains the original Stage 4 semantic representation while integrating multi-scale local context with global spatial dependencies, and serves as the global feature input to the cross-layer fusion module.

#### 2.2.4. Interactive Cross-Attention Fusion (ICAF) Module

Shallow features in cotton pest and disease images retain fine-grained information such as leaf color, local texture, and edges, whereas deep features provide more abstract semantic representations. Because these feature types differ in channel dimensionality, spatial resolution, and semantic level, direct concatenation or element-wise addition lacks an explicit mechanism for cross-layer interaction. To address this limitation, the Interactive Cross-Attention Fusion (ICAF) module performs bidirectional cross-attention in a shared token space and then reshapes the interaction-enhanced representations back to the spatial feature domain. Its architecture is illustrated in Figure 3c.

ICAF takes the local feature $F_{\mathrm{local}}$ produced by DLE and the global feature $F_{\mathrm{global}}$ produced by G-MSLA. $F_{\mathrm{local}}$ is bilinearly upsampled to 56 × 56 with align_corners = False. The two features are independently flattened, transposed, and linearly projected to an embedding dimension of 512. Learnable 2D positional embeddings are interpolated from a 16 × 16 base grid to 56 × 56 and added to the corresponding token sequences as defined in Equation (9).

(9) $$\begin{array}{l} Z_{l}=P_{l}\left(\mathrm{FlattenTranspose}(F_{\mathrm{local}})\right)+P_{l}\\ Z_{g}=P_{g}\left(\mathrm{FlattenTranspose}\left(Up\left(F_{\mathrm{global}}\right)\right)\right)+P_{g} \end{array}$$

where $P_{l}$ and $P_{g}$ denote linear projections of (256 → 512) and (2048 → 512), respectively. Up(⋅) denotes bilinear upsampling, while $P_{l}$ and $P_{g}$ are the interpolated learnable positional embeddings. Both $Z_{l}$ and $Z_{g}$ have dimensions of *B* × 3136 × 512. Bidirectional interaction is realized through two independent cross-attention blocks, each consisting of multi-head cross-attention followed by a feed-forward network (FFN). In the global-to-local direction, local tokens serve as queries and global tokens provide keys and values, enabling the global semantic representation to guide the refinement of the local representation. In the local-to-global direction, the assignment is reversed, as defined in Equation (10).

(10) $$\begin{array}{l} Y_{l}=\mathrm{CA}\left(Z_{l},Z_{g}\right)\\ Y_{g}=\mathrm{CA}\left(Z_{g},Z_{l}\right) \end{array}$$

The interacted token sequences $Y_{l}$ and $Y_{g}$ are separately reconstructed as feature maps of *B* × 512 × 56 × 56. Each branch is projected to 256 channels via a 1 × 1 convolution followed by batch normalization and ReLU. The two projected features are concatenated along the channel dimension and processed by a fusion Conv-BN-ReLU projection, as defined in Equation (11).

(11) $$\begin{array}{l} R_{l}=\phi_{l}\left(\mathrm{Reshape}\left(Y_{l}\right)\right)\\ R_{g}=\phi_{g}\left(\mathrm{Reshape}\left(Y_{g}\right)\right)\\ F_{\mathrm{fused}}=\phi_{f}\left(\left[R_{l};R_{g}\right]\right) \end{array}$$

where $\phi_{l}$ and $\phi_{g}$ denote 512 → 256 Conv-BN-ReLU projections, and $\phi_{f}$ denotes the 512 → 256 fusion projection. A learnable scalar α controls the contribution of the original local representation through a final residual connection, as defined in Equation (12).

(12) $$F_{\mathrm{ICAF}}=F_{\mathrm{fused}}+\alpha F_{\mathrm{local}},\alpha_{init}=0.1$$

The scalar α is learnable and is not restricted to a predefined range. The output $F_{\mathrm{ICAF}}$ contains bidirectionally interacted local and global representations and is subsequently used for classification.

The modules in IFMA-Net were selected according to four criteria: task-specific visual requirements, the use of established components within a task-oriented framework, quantitative ablation validation, and computational considerations. DLE was applied to the high-resolution output of Stage 1 because local textures and symptom boundaries are more explicitly represented at this stage. G-MSLA was applied to the output of Stage 4 because its compact spatial resolution makes the modeling of multi-scale context and long-range dependencies computationally manageable. ICAF was designed to align and bidirectionally fuse the enhanced shallow and deep representations.

### 2.3. Data Preprocessing, Training, and Implementation Details

All models were evaluated using the same dataset partitions, preprocessing procedure, and training protocol. Images were resized to 224 × 224 pixels and normalized using ImageNet statistics. Each model was trained end-to-end using AdamW for a maximum of 60 epochs. A linear warm-up was applied during the first 5 epochs and subsequently adjusted using cosine annealing. Model selection was based on the validation macro-F1 score. Training was terminated if this score did not improve for 15 consecutive epochs, and the checkpoint with the highest validation macro-F1 score was retained for testing. Label smoothing and FP16 mixed-precision training were also applied. Detailed hyperparameter settings of DLE, G-MSLA, and ICAF are provided in Table S4 (Supplementary Materials).

The ResNet-50 backbone was initialized with ImageNet-pretrained weights, whereas the convolutional and linear weights in DLE, G-MSLA, and ICAF were initialized using Xavier normal initialization. Each model configuration was evaluated in three separate stochastic training runs using fixed random seeds of 42, 123, and 456. These runs represent repeated model-training evaluations rather than biological replicates. The results are reported as the mean ± standard deviation across the three runs. Because only three runs were available, no formal statistical significance test was performed, as such a small sample would provide limited statistical power and potentially unstable inference. Accordingly, the reported differences are interpreted as descriptive differences in mean performance under the specified experimental protocol rather than as statistically significant effects. Accuracy and macro-F1 score were used as classification metrics, while parameter count and FLOPs were reported to characterize model complexity. The complete training configuration is summarized in Table S5 (Supplementary Materials).

### 2.4. Evaluation Indicators

To assess model performance in the multi-class classification of healthy, diseased, and pest-damaged cotton leaves, accuracy. Let N denote the total number of samples in the test set; in this experiment N=447. Let $N_{correct}$ denote the number of correctly predicted samples. Accuracy is defined as shown in Equation (13).

(13) $$Accuracy=\frac{N_{correct}}{N}$$

Accuracy measures overall correctness but may obscure poor performance on minority classes in an imbalanced dataset.

For the i-th class, let ${TP}_{i}$,$\;FP_{i}$, and $FN_{i}$ denote the numbers of true positives, false positives, and false negatives, respectively. ${\mathrm{Precision}}_{i}$, ${\mathrm{Recall}}_{i}$, and ${F1}_{i}$ are defined as shown in Equation (14).

(14) $$\begin{array}{l} {\mathrm{Recall}}_{i}=\frac{TP_{i}}{TP_{i}+FN_{i}}\\ {\mathrm{Precision}}_{i}=\frac{TP_{i}}{TP_{i}+FP_{i}}\\ {F1}_{i}=\frac{{2\times Precision}_{i}\times Recall_{i}}{{Precision}_{i}+Recall_{i}}\\ macro-F1=\frac{1}{C}\sum_{i=1}^{C}F1_{i} \end{array}$$

Together, these metrics assess overall accuracy, per-class recognition performance, label agreement, and computational efficiency in cotton pests and diseases classification.

## 3. Experimental Results and Analysis

IFMA-Net was evaluated through comparisons with representative classification models, module and fusion ablations, cross-source testing, computational-cost analysis, and robustness experiments. Unless otherwise stated, each model configuration was independently trained using random seeds 42, 123, and 456, and the results are reported as the mean ± standard deviation.

### 3.1. Comparison with Representative Baseline Models

Table 1 compares IFMA-Net with conventional convolutional networks, attention-enhanced ResNet-50 variants, lightweight networks, and modern ConvNet and Transformer-based architectures on the eight-class internal test set, using identical data partitions, preprocessing procedures, and evaluation protocols. IFMA-Net achieved the highest mean accuracy and macro-F1 score, at 95.53% and 94.81%, respectively, exceeding ResNet-50 by 5.52 and 7.32 percentage points, respectively. Compared with ConvNeXt-T, the best-performing competing model, IFMA-Net outperformed it by 0.42 and 0.69 percentage points in the two metrics, respectively. However, as these results are based on only three independent runs without statistical significance testing, these small margins indicate higher mean performance but should not be interpreted as statistically significant superiority.

### 3.2. Ablation Experiments

#### 3.2.1. Effects of Individual Modules and Module Combinations

Table 2 reports the effects of DLE, G-MSLA, and ICAF using ResNet-50 as the baseline. The baseline achieved an accuracy of 90.01% and a macro-F1 score of 87.49%. When introduced individually, DLE, G-MSLA, and ICAF achieved mean accuracies of 92.15%, 91.08%, and 92.10%, respectively, while their macro-F1 scores ranged from 90.01% to 91.49%. Among the single-module configurations, DLE achieved the highest mean accuracy, whereas ICAF achieved the highest mean macro-F1 score. For the dual-module configurations, mean accuracies ranged from 93.02% to 93.19% and macro-F1 scores from 92.58% to 93.52%, exceeding the best single-module results under the same protocol. These results indicate that combining the modules yields higher mean performance. However, because only three independent runs were performed, no formal significance test was conducted, and the results do not establish statistical significance or a general causal relationship among the modules. In particular, the performance gaps between the last two configurations (Concat/Add residual fusion and the proposed learnable residual fusion) are small and should not be regarded as conclusive.

The ablation results in Table 2 support the contribution of these modules under the present experimental protocol, while the complexity analysis in Section 3.4 reports the associated computational costs.

#### 3.2.2. Comparison of Multi-Module Fusion Strategies

As shown in Table 2, the three-module configurations were further compared using different final aggregation strategies. Bias residual fusion achieved an accuracy of 93.25% and a macro-F1 score of 92.64%, whereas Concat/Add residual fusion outperformed it with scores of 95.08% and 94.68%, respectively. The proposed learnable residual fusion yielded the highest performance, with an accuracy of 95.53% and a macro-F1 score of 94.81%. Relative to Concat/Add fusion, these improvements were only 0.45 and 0.13 percentage points in accuracy and macro-F1, respectively. No formal significance test was performed owing to the limited number of runs (*n* = 3); therefore, the results support the selected fusion strategy under the present experimental setting but do not guarantee its universal effectiveness.

#### 3.2.3. Class-Wise Performance Analysis

To examine how the ablation-related performance differences were distributed across categories, Figure 4 presents the class-wise F1 scores of ResNet-50, ResNet-50+DLE, ResNet-50+DLE+G-MSLA, and IFMA-Net. The values were averaged over the three random seeds, and the error bars represent the corresponding standard deviations.

ResNet-50 obtained F1 scores of 64.1% for Fusarium Wilt and 80.7% for Leaf Spot. Adding DLE increased the F1 score of Fusarium Wilt to 82.5%. The effects of the subsequent modules varied across classes rather than producing uniform gains. IFMA-Net achieved the highest or comparable mean F1 score for most categories. The Grey Mildew and Leaf Curl classes contained only 19 and 21 internal-test samples, respectively; their estimates are consequently sensitive to a small number of prediction changes and should be interpreted cautiously.

#### 3.2.4. Confusion-Matrix Analysis

To examine class-specific prediction patterns, Figure 5 presents the row-normalized mean confusion matrices of ResNet-50 and IFMA-Net on the eight-class internal test set. The matrices from the three random-seed runs were aggregated using the procedure specified in the figure caption. Rows denote true classes, columns denote predicted classes, and diagonal entries correspond to class recall. Displayed percentages were rounded to integers; therefore, some row totals may differ slightly from 100%.

Compared with ResNet-50, IFMA-Net showed higher diagonal values for most classes. The recall of Fusarium Wilt increased from 54% to 83%, while that of Leaf Spot increased from 83% to 90%. Under the present test conditions, IFMA-Net therefore reduced the observed misclassification proportions for these two classes. The matrices characterize class-level prediction behavior and do not provide evidence of lesion localization.

### 3.3. Cross-Source Evaluation on an Independent Public Dataset

Cross-source performance was evaluated using the official test split of the Cotton Crop Diseases dataset (Roboflow Universe). Four classes common to both datasets were selected: Healthy, Aphids, Armyworm, and Bacterial Blight, yielding an external test set of 437 images held out from training and all model-selection procedures. All models were trained on the eight-class training split of the self-constructed dataset; internal metrics were computed on the four shared classes of the internal test set, with the same models tested directly on the external subset.

As shown in Table 3, IFMA-Net achieved an internal accuracy of 95.28% and a macro-F1 score of 91.26%. On the external test set, its accuracy and macro-F1 score were 92.36% and 90.21%, respectively. In terms of external macro-F1, IFMA-Net outperformed ResNet-50, Swin-T, and ConvNeXt-T by 8.71, 7.14, and 2.16 percentage points, respectively, and achieved the smallest internal-to-external macro-F1 drop (1.05 percentage points) among the evaluated models. These results indicate favorable cross-source performance relative to the competing models on this test set, while broader domain generalization requires further validation with additional independent datasets.

### 3.4. Analysis of Computational Overhead and Efficiency

In addition to classification accuracy, model size is critical for practical deployment. IFMA-Net achieved the highest mean accuracy of 95.53% but also the largest parameter count of 45.20 M among the evaluated models. Compared with ResNet-50 (23.52 M, 90.01%), IFMA-Net improved accuracy by 5.52 percentage points while nearly doubling the parameter count. Across the evaluated models, larger architectures tended to achieve higher accuracy, whereas the lightweight models (MobileNetV3-Large and ShuffleNetV2) remained competitive with substantially fewer parameters and lower GFLOPs. These results indicate an accuracy–efficiency trade-off rather than a universally optimal choice. They do not by themselves establish suitability for resource-constrained deployment; such deployment would require model compression and hardware-specific validation. This trade-off is visualized in Figure S1 (Supplementary Materials).

### 3.5. Robustness Under Controlled Noise and Occlusion

To evaluate the stability of IFMA-Net under complex field conditions, two representative disturbances, Gaussian noise and random occlusion, were introduced at three intensity levels.

#### 3.5.1. Overall Robustness Evaluation Under Noise and Occlusion Perturbations

Gaussian noise (zero mean; σ = 0.05, 0.10, and 0.20 on normalized [0, 1] pixels, clipped after addition) and random square occlusion (10%, 20%, and 30% area coverage; occluded pixels set to zero) were applied online to the clean internal test images at three intensity levels, denoted L1, L2, and L3, respectively. All random seeds were fixed for reproducibility, and models were trained exclusively on clean images without fine-tuning on perturbed samples.

As shown in Table 4, macro-F1 decreased for both models as intensity increased. Under random occlusion, IFMA-Net declined much less than ResNet-50 at higher occlusion levels, outperforming ResNet-50 by 9.69 percentage points (89.22 vs. 79.53) at L1, by 24.23 percentage points (83.52 vs. 59.29) at L2, and by 35.00 percentage points (73.35 vs. 38.35) at L3; The retention rates were 3.20, 20.32, and 33.54 percentage points higher at L1, L2, and L3, respectively. Under Gaussian noise, IFMA-Net was slightly higher than ResNet-50 at L1 (90.18 vs. 85.96). Although both models declined at L2 and L3, IFMA-Net remained slightly higher (77.95 vs. 71.03 at L2; 53.62 vs. 49.18 at L3), with both falling below 55% at L3. Retention rates were slightly higher for ResNet-50 at L1 (98.25 vs. 95.12), and both fell to similar levels at L2 and L3. These results indicate that IFMA-Net exhibited greater robustness to occlusion but only modest gains under severe noise, and they should not be extrapolated to other forms of field degradation.

#### 3.5.2. Analysis of Category Robustness Under High-Intensity Noise

Under L3 Gaussian noise, class-wise accuracy varied substantially, indicating uneven sensitivity to severe noise. As shown in Figure S2 (Supplementary Materials), IFMA-Net achieved higher accuracy than ResNet-50 for most classes. For Fusarium Wilt, accuracy increased from 17% to 55%, corresponding to an absolute gain of 38 percentage points. Accuracy also improved for Armyworm and Leaf Curl. These results describe the relative performance of the two complete models under the specified synthetic noise condition.

### 3.6. Module-Wise Response Visualization

To evaluate the interpretability of IFMA-Net, Gradient-weighted Class Activation Mapping++ (Grad-CAM++) was adopted to visualize internal feature responses and spatial activation patterns at different network hierarchical levels. Representative pest and disease samples were used for module-level visualization, and difficult samples were further compared among IFMA-Net, ResNet-50, and Swin-T.

#### 3.6.1. Internal Feature Evolution and Decision Regions

To examine whether IFMA-Net focused on biologically relevant regions in cotton field images, Grad-CAM++ was applied to internal feature maps. Representative samples, including Leaf Spot and Armyworm damage, were selected to compare the responses of the original images, the ResNet-50 baseline, and the three proposed IFMA-Net modules, as shown in Figure 6a.

For the selected examples, ResNet-50 produced relatively dispersed response maps, whereas the responses associated with the IFMA-Net branches showed different spatial distributions. DLE exhibited responses around local texture and boundary regions, while G-MSLA produced broader contextual responses. The final ICAF representation combined information from the two branches and showed more concentrated activations around visually apparent symptom or pest regions in some examples. Because lesion-level annotations were unavailable, these qualitative observations cannot establish localization accuracy or confirm that every activated region corresponds to a causal disease feature.

#### 3.6.2. Qualitative Comparison on Challenging and Failure Cases

To assess decision stability under challenging conditions, difficult samples with high inter-class similarity were evaluated using IFMA-Net, ResNet-50, and Swin-T. The corresponding recognition results and attention heatmaps are shown in Figure 6b.

In several representative cases, IFMA-Net highlighted symptom regions that aligned more closely with visually apparent lesions than those identified by ResNet-50 or Swin-T. This observation was purely qualitative and does not constitute a formal localization metric. Misclassification remained frequent when classes exhibited similar visual characteristics, such as necrosis and chlorosis, suggesting that the current image-level supervision was insufficient to fully distinguish some fine-grained categories.

## 4. Discussion

Images of cotton pests and diseases acquired under natural field conditions may exhibit low symptom saliency, cluttered backgrounds, and substantial variation in lesion scale [27], posing significant challenges for vision-based agricultural sensing systems. IFMA-Net was developed to address these sensing conditions by integrating local feature enhancement, multi-scale contextual modeling, and cross-layer feature fusion. This section discusses the observed performance gains, robustness, interpretability, limitations, and potential applications of the proposed architecture in the context of field-deployable agricultural sensing.

### 4.1. Analysis of the Performance Improvement Mechanism

On the eight-class internal test set, IFMA-Net achieved an accuracy of 95.53 ± 0.17% and a macro-F1 score of 94.81 ± 0.14%, surpassing ResNet-50 by 5.52 and 7.32 percentage points, respectively, and yielding the highest mean performance among all compared models. The ablation results showed that DLE, G-MSLA, and ICAF individually improved mean classification performance, while their complete integration produced the highest mean accuracy and macro-F1 score. These results support the complementarity of local enhancement, multi-scale modeling, and cross-layer interaction.

Class-wise results showed that Fusarium Wilt and Leaf Spot were more difficult for ResNet-50 to recognize, possibly because of subtle symptoms and inter-class visual similarity. The response maps were consistent with the intended functions of the proposed modules: DLE preserved shallow texture responses, G-MSLA incorporated multi-scale contextual information, and ICAF facilitated information exchange between shallow and deep features. However, confirming these mechanisms quantitatively would require lesion-level annotations or controlled subgroup experiments.

### 4.2. Comparative Analysis with Existing Methods

IFMA-Net achieved higher mean accuracy and macro-F1 scores than the evaluated convolutional, attention-enhanced, lightweight, and Transformer-based models. Conventional convolutional networks may be affected by irrelevant background features when symptoms are subtle or have low contrast [28,29]. Unlike single attention operations, IFMA-Net combines local enhancement, contextual modeling, and bidirectional cross-layer interaction.

Compared with ConvNeXt-T, the strongest competing model, IFMA-Net increased accuracy and macro-F1 by 0.42 and 0.69 percentage points, respectively. Because we conducted only three repeated runs and did not perform inferential statistical tests, these differences reflect higher average performance rather than statistically verified superiority. In addition, IFMA-Net used more parameters and GFLOPs, indicating a trade-off between performance and computational complexity that is particularly relevant for resource-constrained sensing devices such as smartphones and edge computing platforms deployed in the field.

On the independent public dataset, IFMA-Net showed a smaller macro-F1 decrease from the internal to the external test set than the competing models, indicating improved stability under the evaluated source shift. However, the external experiment included only four shared classes from a single public dataset and therefore did not establish generalization across arbitrary regions, years, or acquisition devices.

### 4.3. Analysis of Robustness and Interpretability

Under Gaussian noise and random occlusion, IFMA-Net generally maintained higher raw macro-F1 scores than ResNet-50. The advantage was more pronounced under random occlusion, which simulates common field sensing artifacts such as partial leaf coverage by adjacent foliage or imperfect sensor framing. In contrast, severe Gaussian noise, which may arise from low-light imaging or high sensor gain in overcast or dusk conditions, caused substantial performance degradation in both models. These perturbations represent selected forms of degradation and did not fully reproduce field variations such as uneven illumination, overlapping leaves, and background clutter [30].

Grad-CAM++ visualizations showed that IFMA-Net generated more sharply focused, class-discriminative responses over symptomatic leaf regions in the presented examples. However, because lesion-level annotations were not available, these results provided only qualitative insight into the response distributions and did not allow a rigorous evaluation of localization accuracy.

### 4.4. Limitation Analysis

The self-constructed dataset contained 5400 images and exhibited class imbalance, which may affect minority-class estimates. From a sensing perspective, the dataset was acquired using specific smartphone and camera models under controlled time windows; performance may vary when captured with different sensor hardware, lens configurations, or extreme weather conditions. Differences in acquisition conditions, category definitions, and class distributions between the self-constructed and public dataset may also introduce domain shift [31,32]. The external evaluation included only four shared classes, and three repeated runs provided limited statistical power. IFMA-Net also incurred higher computational costs than the competing models, which may restrict deployment on resource-constrained sensing devices such as field-deployed edge nodes; knowledge distillation could be explored in future work to compress the model while preserving accuracy. In addition, the absence of lesion-level annotations prevented quantitative localization and severity assessment. Moreover, the disease-related categories were assigned based on field-visible symptoms and expert agronomic review, without laboratory confirmation of the collected field samples. Because different biotic or abiotic factors may produce overlapping symptoms, some labels may involve etiological misclassification. Laboratory confirmation of a representative subset of samples will be considered in future dataset development to improve the etiological reliability of the category labels.

## 5. Conclusions

IFMA-Net, built on ResNet-50, addresses fine-grained symptom recognition, low contrast, leaf folding and mutual occlusion, scale variation, and background interference in cotton field images, offering a task-oriented solution for vision-based agricultural sensing. The DLE module preserves shallow texture and boundary information, G-MSLA combines multi-scale dilated depthwise separable convolutions with self-attention to model both local and global context, and ICAF employs two oppositely directed cross-attention branches to achieve local–global interaction and learnable residual fusion. The primary contribution lies in the task-oriented hierarchical integration of established operations, rather than in introducing a fundamentally new attention mechanism.

IFMA-Net yielded the highest mean performance among all compared models for cotton pest and disease identification. Ablation experiments confirmed that all three proposed modules contributed to classification performance, although their combined effect depended on the selected feature-fusion strategy. Class-level results showed fewer errors in several easily confused categories. External evaluation demonstrated cross-source generalization but also revealed a noticeable domain shift. IFMA-Net remained relatively robust to random occlusion, but performance degraded under severe Gaussian noise. Visualizations provided qualitative comparisons of response distributions but did not establish precise lesion localization.

IFMA-Net improved cotton pest and disease classification under the reported dataset and experimental conditions. However, its parameter count, computational cost, and inference latency may limit direct deployment on resource-constrained devices. Future work will evaluate cross-year, cross-location, and cross-device generalization; investigate few-shot learning and domain adaptation for underrepresented categories; and apply knowledge distillation, pruning, and quantization to enable efficient deployment on edge computing platforms and smartphone-based field sensing systems. Lesion-level detection or segmentation annotations will also be introduced to quantify spatial-response performance and support disease-severity estimation.

## Figures and Tables

**Figure 1:**
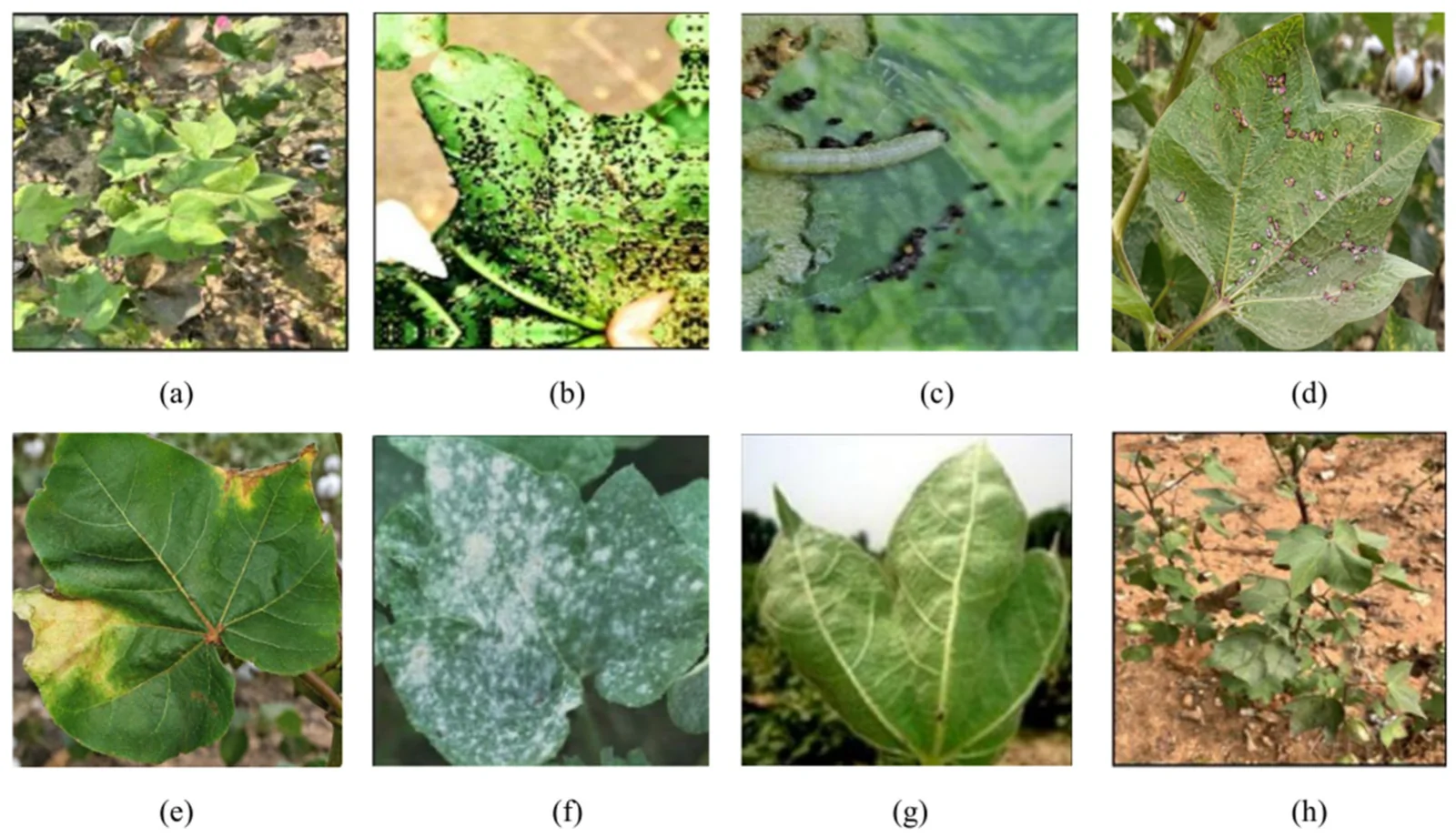
Representative samples of the eight classes in the cotton pest and disease dataset. (**a**) Healthy; (**b**) Aphids; (**c**) Armyworm; (**d**) Bacterial Blight; (**e**) Fusarium Wilt; (**f**) Grey Mildew; (**g**) Leaf Curl; (**h**) Leaf Spot. Because the original field photographs were collected without calibrated spatial references, Figure 1 is presented for category and symptom illustration only. Leaf, pest, and lesion dimensions were not quantitatively interpreted from these images.

**Figure 2:**
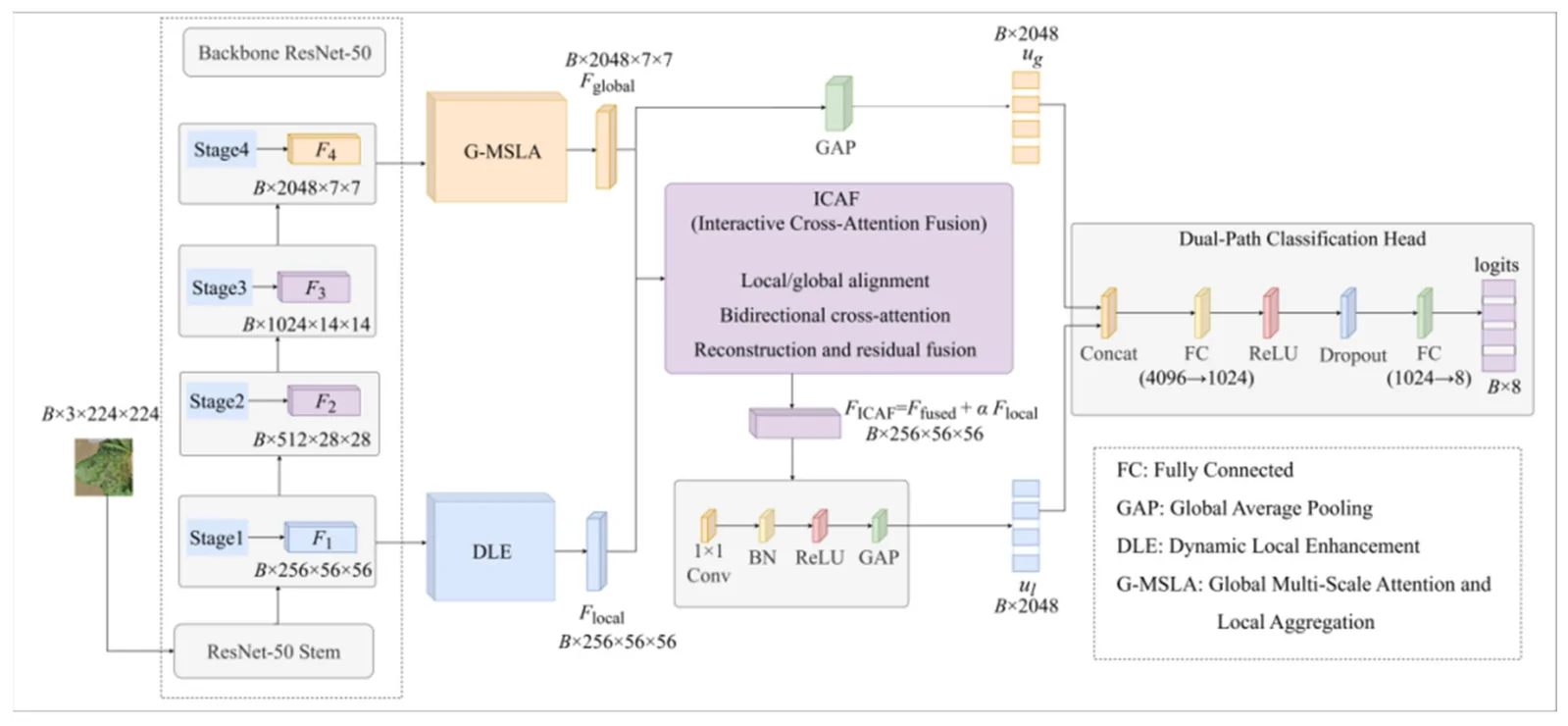
Overall architecture of IFMA-Net. A ResNet-50 backbone extracts shallow $F_{1}$ and deep $F_{4}$ features, which are enhanced in parallel by DLE and G-MSLA, fused by ICAF via bidirectional cross-attention, and classified by a dual-path head for eight-class prediction.

**Figure 3:**
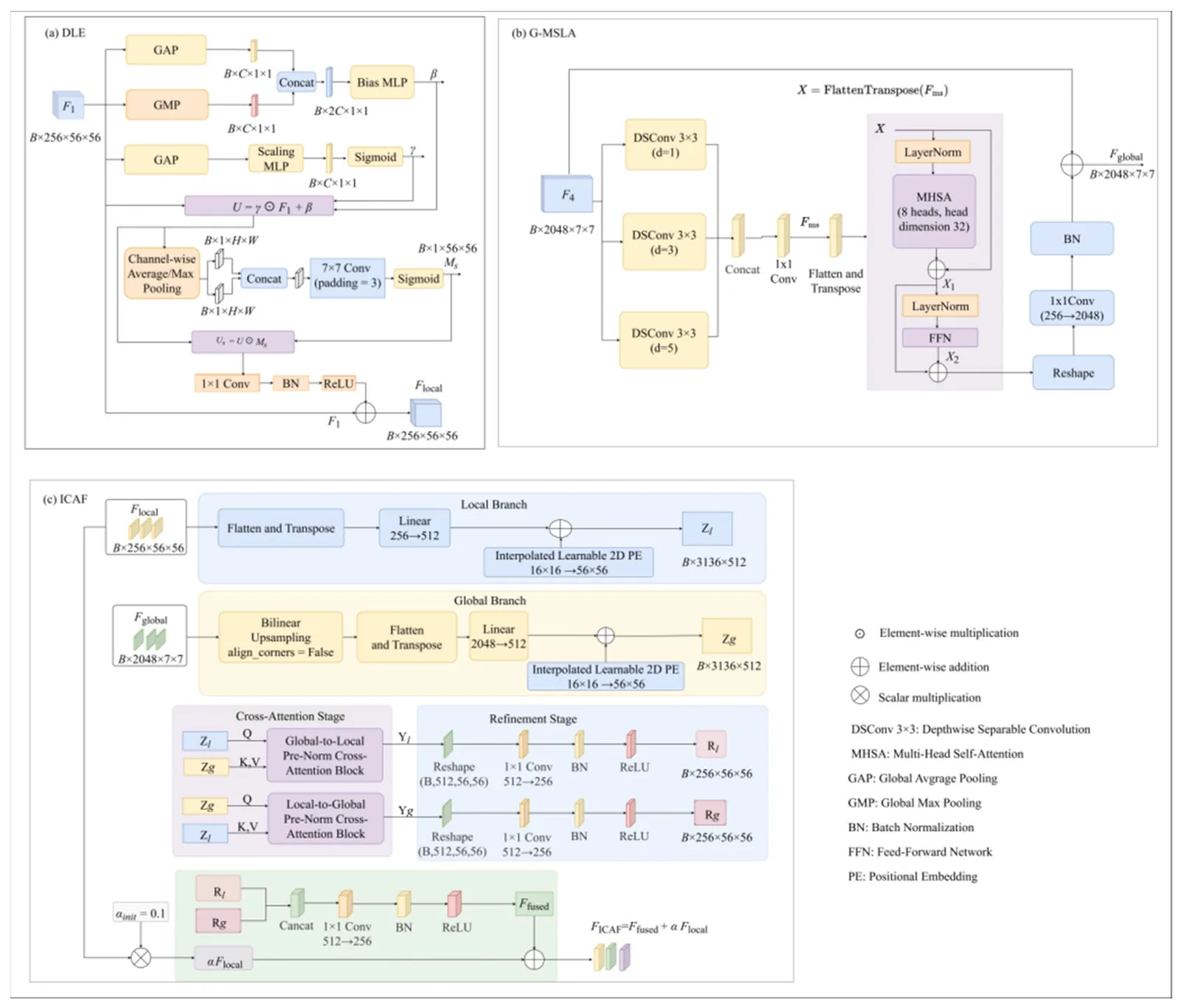
Detailed architectures of the proposed modules. (**a**) Dynamic Local Enhancement (DLE), (**b**) Global Multi-Scale Attention and Local Aggregation (G-MSLA), and (**c**) Interactive Cross-Attention Fusion (ICAF). DLE enhances shallow local features through channel-wise affine modulation and spatial attention. G-MSLA captures multi-scale contextual information using dilated depthwise separable convolutions and a Pre-Norm multi-head self-attention block. ICAF aligns local and global representations through bidirectional cross-attention and integrates the refined features using learnable residual fusion.

**Figure 4:**
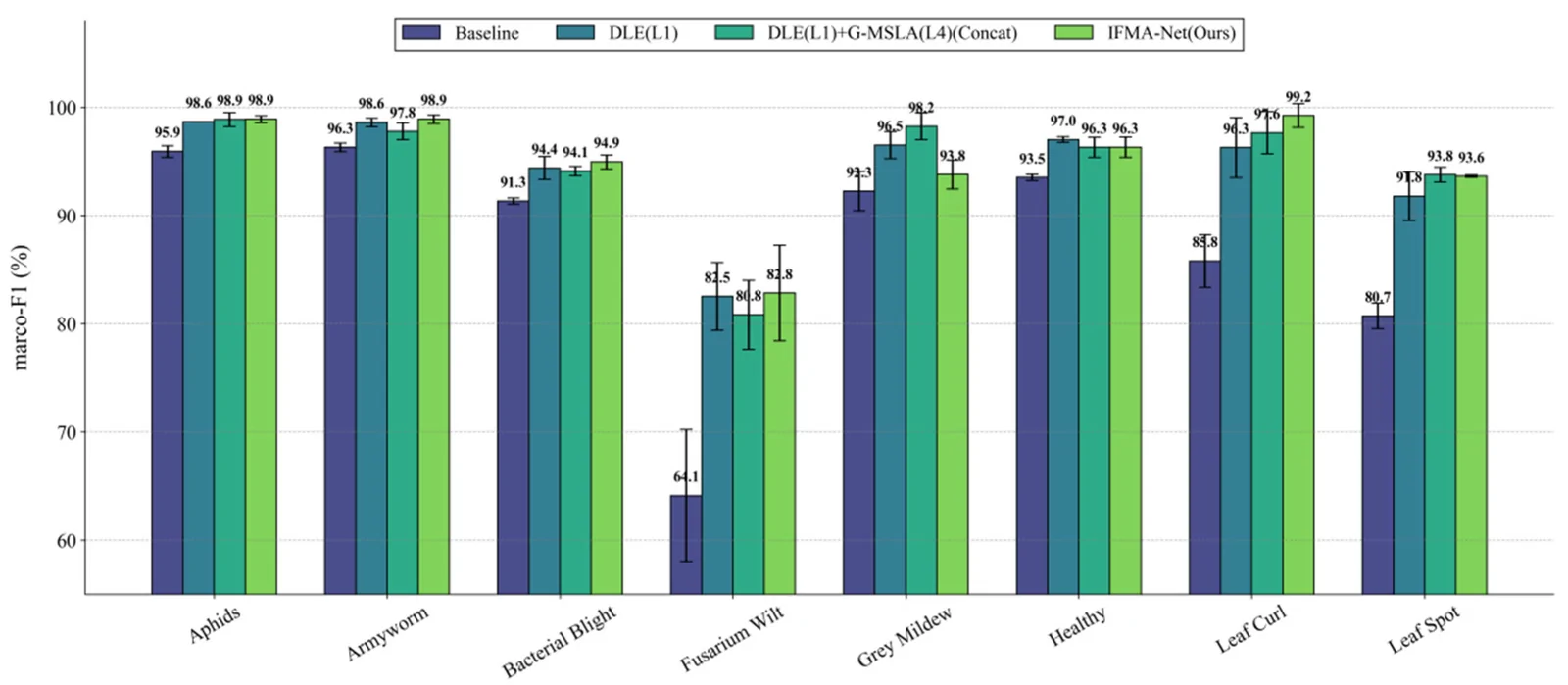
Class-wise F1 scores of four model configurations on the eight-class internal test set. Bars represent means over three runs with random seeds 42, 123, and 456, and error bars denote standard deviations.

**Figure 5:**
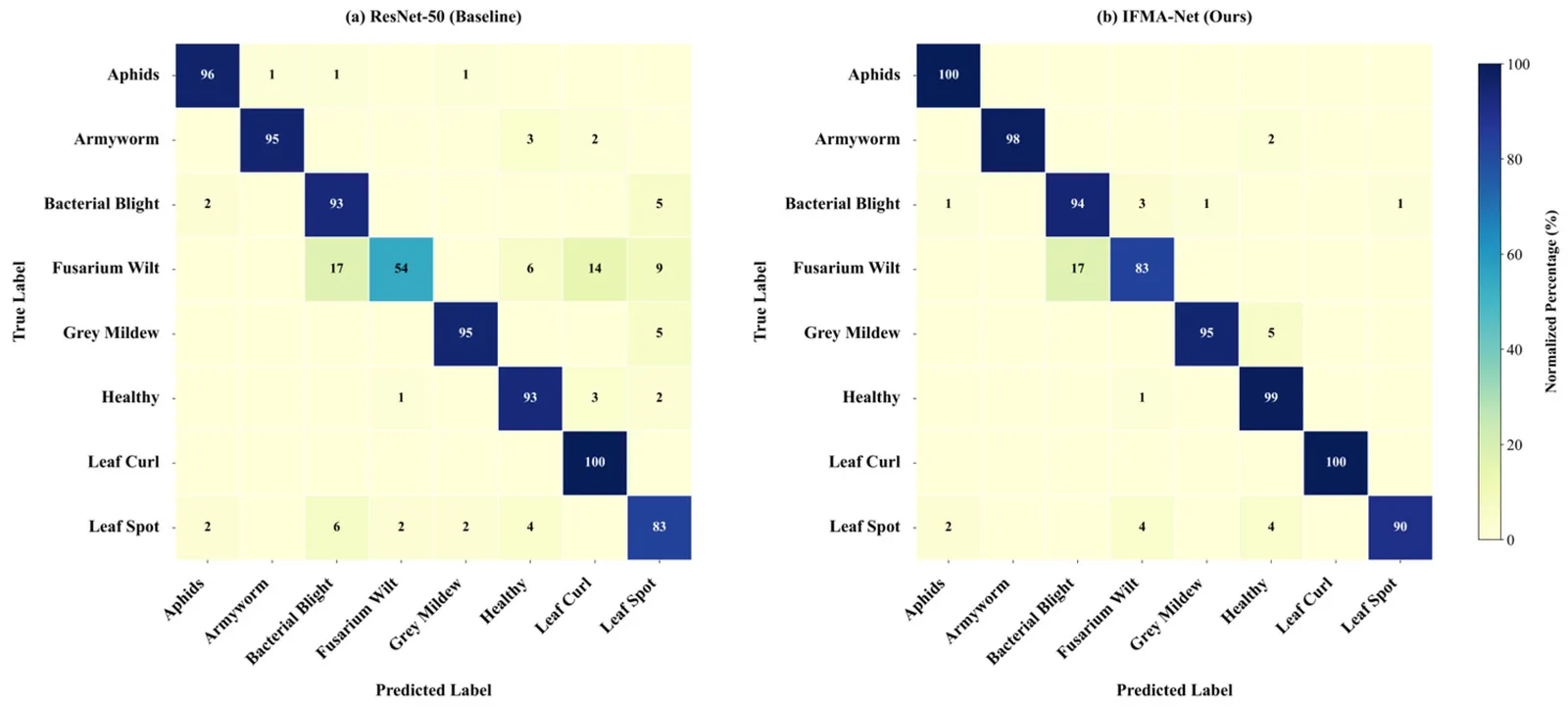
Row-normalized mean confusion matrices of ResNet-50 and IFMA-Net on the internal eight-class test set. (**a**) ResNet-50, (**b**) IFMA-Net. A unified color scale is applied for both subplots. Minor deviations from 100% in row sums are caused by integer rounding.

**Figure 6:**
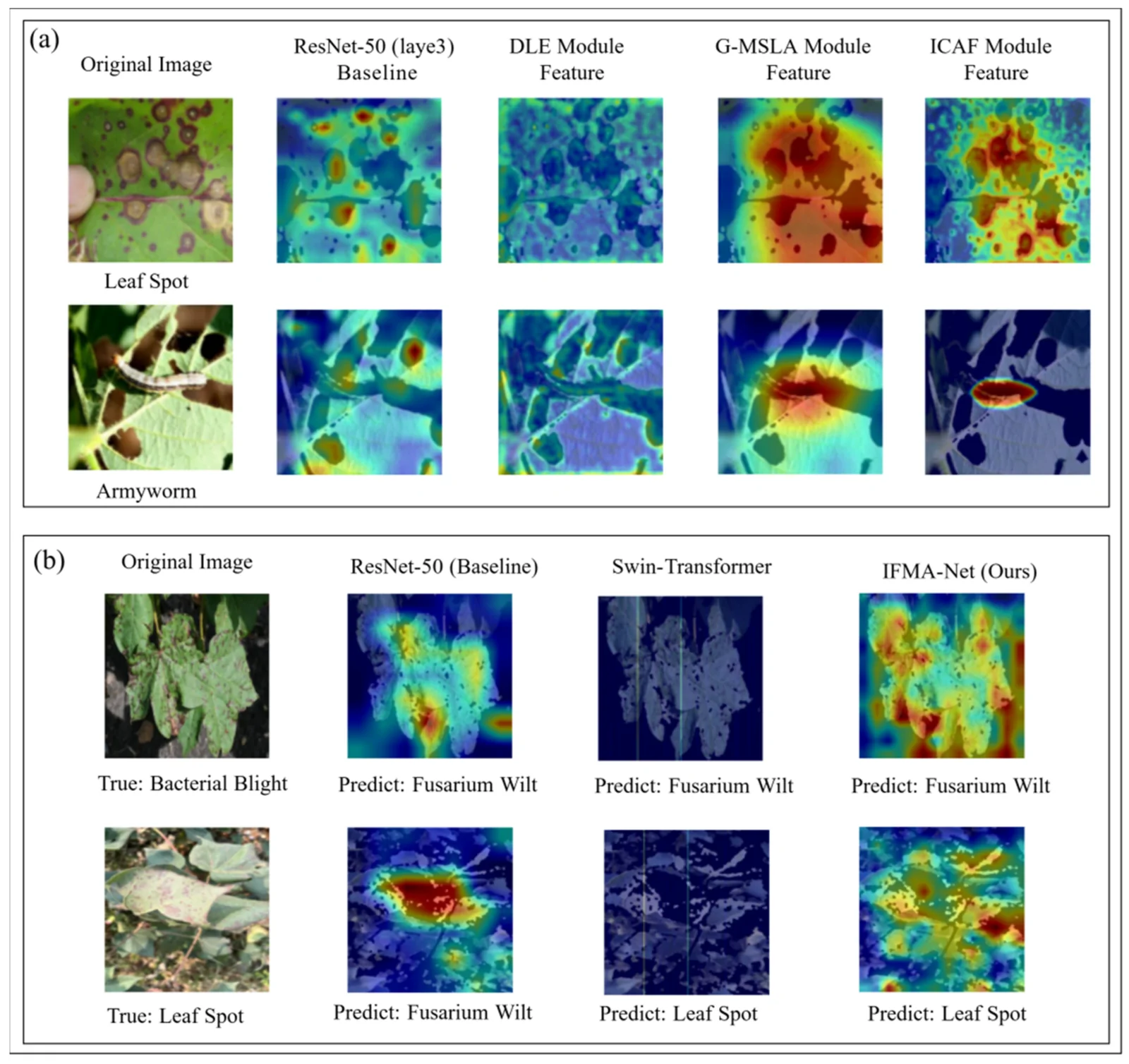
Grad-CAM++ visualization of internal feature responses and challenging prediction cases. (**a**) Grad-CAM++ response maps obtained from the ResNet-50 baseline and the intermediate outputs associated with DLE, G-MSLA, and ICAF for selected cotton pest and disease images. (**b**) Grad-CAM++ response maps and predictions of ResNet-50, Swin-T, and IFMA-Net for selected challenging samples. Warmer colors indicate stronger model responses, whereas cooler colors indicate weaker responses. These visualizations provide qualitative insight into model behavior and should not be interpreted as quantitative evidence of localization accuracy.

**Table 1:** Performance and computational-cost comparison on the eight-class internal test set.

Model	Accuracy (%)	Macro-F1 (%)	Params (M)	GFLOPs
ResNet-50	90.01 ± 0.56	87.49 ± 0.73	23.52	6.57
ResNet-50 + CBAM(Full)	91.80 ± 0.04	90.44 ± 0.41	24.22	6.68
ResNet-50 + SE(Full)	94.90 ± 0.21	93.39 ± 0.27	24.22	6.68
ResNet-50 + SE(Shallow)	94.63 ± 0.66	93.78 ± 0.50	23.57	6.59
Swin-T	95.02 ± 0.18	94.07 ± 0.35	27.53	10.40
MobileNetV3-Large	92.15 ± 0.42	91.03 ± 0.51	5.48	0.32
ShuffleNetV2	91.68 ± 0.53	90.76 ± 0.62	2.28	0.18
EfficientNet-B4	94.26 ± 0.33	93.51 ± 0.46	19.16	12.50
ConvNeXt-T	95.11 ± 0.25	94.12 ± 0.31	28.62	8.50
IFMA-Net (Ours)	95.53 ± 0.17	94.81 ± 0.14	45.20	19.85

Note: Accuracy and macro-F1 are reported as mean ± standard deviation over three independent training runs using random seeds 42, 123, and 456. These runs represent repeated stochastic training evaluations rather than biological replicates. Parameter count and GFLOPs are deterministic model-complexity measures. Because only three runs were conducted, differences in classification performance are presented descriptively and should not be interpreted as statistically significant.

**Table 2:** Ablation results for the proposed modules and final aggregation strategies.

DLE	G-MSLA	ICAF	Final Aggregation	Accuracy (%)	Macro-F1 (%)
—	—	—	—	90.01 ± 0.56	87.49 ± 0.73
√	—	—	—	92.15 ± 0.69	91.46 ± 0.88
—	√	—	—	91.08 ± 0.66	90.01 ± 0.95
—	—	√	Learnable residual aggregation	92.10 ± 0.48	91.49 ± 0.67
√	√	—	Concat + 1 × 1 Conv	93.02 ± 0.45	92.58 ± 0.51
√	—	√	Learnable residual aggregation	93.15 ± 0.38	93.37 ± 0.37
—	√	√	Learnable residual aggregation	93.19 ± 0.42	93.52 ± 0.49
√	√	√	Bias residual fusion	93.25 ± 0.25	92.64 ± 0.18
√	√	√	Concat/Add residual fusion	95.08 ± 0.48	94.68 ± 0.64
√	√	√	Proposed learnable residual fusion	95.53 ± 0.17	94.81 ± 0.14

Note: Accuracy and macro-F1 are reported as mean ± standard deviation over three independent training runs using random seeds 42, 123, and 456. The experiments used the same dataset partition and training protocol. These runs are not biological replicates. No formal significance test was conducted because of the limited number of runs; therefore, the reported differences represent descriptive mean performance under the present experimental setting. A checkmark (√) indicates that the corresponding module is enabled, and the em dash (—) means the module is not adopted.

**Table 3:** Cross-source performance on the internal and independent public test set.

Model	Internal Acc (%)	Internal Macro-F1 (%)	External Acc (%)	External Macro-F1 (%)	F1 Drop (pp)
ResNet-50	90.28 ± 0.53	86.64 ± 0.08	89.96 ± 0.38	81.50 ± 0.09	5.14
Swin-T	91.74 ± 0.47	87.91 ± 0.15	90.40 ± 0.46	83.07 ± 0.64	4.84
ConvNeXt-T	92.94 ± 0.45	90.72 ± 0.26	91.09 ± 0.58	88.05 ± 0.41	2.67
IFMA-Net(Ours)	95.28 ± 0.14	91.26 ± 0.05	92.36 ± 0.17	90.21 ± 0.24	1.05

Note: All models were trained on the eight-class training subset of the self-constructed dataset and evaluated only on the four classes shared with the external dataset; predictions on non-shared classes were counted as incorrect. “Internal” denotes the shared classes in the internal test set, and “External” denotes the 437-image official test subset of the Roboflow dataset. F1 drop = Internal macro-F1 − External macro-F1; a larger value indicates greater cross-source performance degradation.

**Table 4:** Robustness retention rates of ResNet-50 and IFMA-Net under different perturbations.

Perturbation Type	Level	ResNet-50 Macro-F1 (%)	IFMA-Net Macro-F1 (%)	ResNet-50 Retention (%)	IFMA-Net Retention (%)
Random Occlusion	L1	79.53	89.22	90.90	94.10
(10%/20%/30% area)	L2	59.29	83.52	67.77	88.09
	L3	38.35	73.35	43.83	77.37
Gaussian Noise	L1	85.96	90.18	98.25	95.12
(σ = 0.05/0.10/0.20)	L2	71.03	77.95	81.19	82.22
	L3	49.18	53.62	56.21	56.56

Note: L1–L3 denote increasing perturbation intensities: Random occlusion covering 10%, 20%, and 30% of the image area with zero-filled square regions. Gaussian noise with σ = 0.05, 0.10, and 0.20 on normalized [0, 1] pixels; The retention rate is defined as (perturbed macro-F1/clean macro-F1) × 100%. The clean macro-F1 scores are 87.49% for ResNet-50 and 94.81% for IFMA-Net.

## Data Availability

The publicly available dataset used in this study is accessible at https://universe.roboflow.com/deep-learning-nygzt/cotton-crop-dieases (accessed on 25 October 2025). The self-built field image dataset of cotton pests and diseases is collected in cooperation with Xinjiang Huier Zhilian Co., Ltd. (Changji, Xinjiang, China). Due to the confidentiality constraints of the enterprise cooperation agreement, this dataset cannot be publicly released. Relevant data can be obtained from the corresponding author upon reasonable request.

## Supplementary Materials

The following supporting information can be downloaded at: https://www.mdpi.com/article/10.3390/s26196026/s1, Table S1: Symptomatology and commonly associated pests or putative causal agents of the eight classes in the cotton pest and disease dataset. Table S2: Class distribution statistics of the cotton pest and disease dataset. Table S3: Category distribution of independent public external test set. Table S4: Parameter settings of DLE, G-MSLA and ICAF modules. Table S5: Training settings and implementation details. Figure S1: Accuracy–complexity comparison. Left: accuracy vs. parameters (M); right: accuracy vs. inference latency (ms). The dashed line marks the 5.52-percentage-point accuracy gap between ResNet-50 and IFMA-Net. Latency was measured on an NVIDIA RTX 4090 (batch size = 1, FP32), averaged over three runs. Figure S2: Class-wise accuracy of ResNet-50 and IFMA-Net under L3 Gaussian noise. Bars show means over three runs using random seeds 42, 123, and 456. Figure S3: Representative field symptom examples of cotton Bacterial Blight and Fusarium Wilt. Images are for symptom illustration only.
