# Supplementary material for: IFMA-Net: Interactive Feature Modulated Attention Network for Accurate Identification of Cotton Pests and Diseases from Field Images
Source: Sensors (Basel). 2026 Sep 23;26(19):6026. doi: 10.3390/s26196026 (PMC13667185; doi:10.3390/s26196026)
Supplement: Supplementary file 1 [file sensors-26-06026-s001.zip › sensors-4555630-supplementary.pdf]

Supplementary

Title: IFMA-Net: Interactive Feature Modulated Attention Network for Accurate Identification of Cotton Pests and Diseases from Field Images

Authors: Lili Song, Zhenxue Chen, Chengyun Liu, Yixin Guo and Dongke Wang

Table S1. Symptomatology and commonly associated pests or putative causal agents of the eight classes in the cotton pest and disease dataset.

Class	commonly associated pest or putative causal agent	Field-visible symptomatology
Healthy	—	Generally uniform green appearance, without obvious visible chlorosis, necrosis, deformation, or pest-related damage.
Aphids	Aphis gossypii Glover (Hemiptera: Aphididae)	Colonies of small, soft-bodied insects commonly observed on the abaxial leaf surface or tender shoots; leaf curling, honeydew deposition, and sooty mold may also occur.
Armyworm	Noctuid lepidopteran larvae, potentially including Helicoverpa armigera and Spodoptera litura	Irregular feeding holes, marginal notches, or partially skeletonized leaves; frass or visible larvae may occasionally be present. Species-level identification cannot be reliably established from feeding damage in field images alone.
Bacterial blight	Xanthomonas citri pv. malvacearum	Angular or vein-limited, water-soaked lesions that may subsequently become dark brown to black. These visible symptoms may overlap with those produced by other biotic or abiotic factors.
Fusarium wilt	Fusarium oxysporum f. sp. vasinfectum	Progressive yellowing, loss of turgor, and wilting, which may appear asymmetric. These above-ground symptoms are not etiologically specific and may overlap with other vascular or abiotic stresses.
Grey mildew	*Ramulariopsis gossypii* (commonly associated)	Pale-green to yellow, sometimes angular lesions, potentially accompanied by white-to-grey superficial growth, particularly on the abaxial leaf surface, and premature defoliation at advanced stages.
Leaf curl (CLCuD)	Begomovirus complex, commonly associated with cotton leaf curl disease; transmitted by Bemisia tabaci	Upward or downward leaf curling, vein thickening, and possible enations on the abaxial surface. These symptoms may vary with disease stage and field conditions.
Leaf spot	Alternaria spp., Cercospora spp., or other putative causes	Circular to irregular necrotic spots, sometimes with concentric rings, pale centers, or chlorotic margins. Similar spot-like symptoms may arise from multiple biotic or abiotic factors.

Note: The classes were assigned according to field-visible symptoms and expert agronomic review. Laboratory confirmation was not performed for the collected field samples. Therefore, the listed pests and putative causal agents are those commonly associated with the observed symptom patterns and should not be interpreted as confirmed identifications for individual images. Some categories may involve etiological misclassification because visually similar symptoms can be produced by different biotic or abiotic factors. “Healthy” denotes an apparently healthy visual category and does not exclude latent or asymptomatic infection. CLCuD, cotton leaf curl disease.

Table S2 Class distribution statistics of the cotton pest and disease dataset

Class	Category	Train	Val	Test	Total
Healthy	Healthy	800	288	89	1177
Aphids	Pest	537	167	74	778
Armyworm	Pest	507	121	60	688
Bacterial Blight	Disease	754	215	101	1070
Fusarium Wilt	Disease	268	100	35	403
Grey Mildew	Disease	154	52	19	225
Leaf Curl	Disease	218	51	21	290
Leaf Spot	Disease	546	175	48	769
Total	—	3784	1169	447	5400

Table S3 Category distribution of independent public external test set

Class	External test samples
Healthy	109
Aphids	99
Armyworm	122
Bacterial Blight	107

Note: This public dataset was obtained from the Roboflow Universe platform (project address: https://universe.roboflow.com/deep-learning-nygzt/cotton-crop-dieases; accessed on October 25, 2025). For cross-domain generalization testing, only four classes that were fully semantically consistent with the self-built dataset were selected.

Table S4 Parameter settings of DLE, G-MSLA and ICAF modules

Module	Hyper-parameter	Setting
DLE	Offset-MLP reduction ratio	16
DLE	Scaling-MLP reduction ratio	4
DLE	Spatial-attention kernel	7 × 7
G-MSLA	Local convolution	3 × 3
G-MSLA	Dilation rates	{1,3,5}
G-MSLA	Embedding dimension	256
G-MSLA	Number of attention heads	8
G-MSLA	FFN expansion ratio	4
ICAF	Alignment method	Bilinear upsampling
ICAF	Embedding dimension	512
ICAF	Number of attention heads	8
ICAF	Positional embedding	Learnable, 16 × 16 base grid
ICAF	Residual coefficient	Learnable scalar,

Note: The embedding dimensions and numbers of attention heads correspond to the actual implementations of G-MSLA and ICAF.

Table S5 Training settings and implementation details

Training setting	Value	Implementation detail	Value
Input size	224×224	GPU	NVIDIA RTX 409
Batch size	8	System Memory	ImageNet pretrained
Initial learning rate	5×10^{-5}	Backbone initialization	64 GB

Minimum learning rate	5×10^{-5}	Proposed-module initialization Training	Xavier normal
Weight decay	5×10^{-4}	Training precision	FP16
Warm-up	5 epochs	Random seeds	42, 123, 456
Early stopping patience	15 epochs	Number of runs	3
Label smoothing	0.1	Optimizer	AdamW

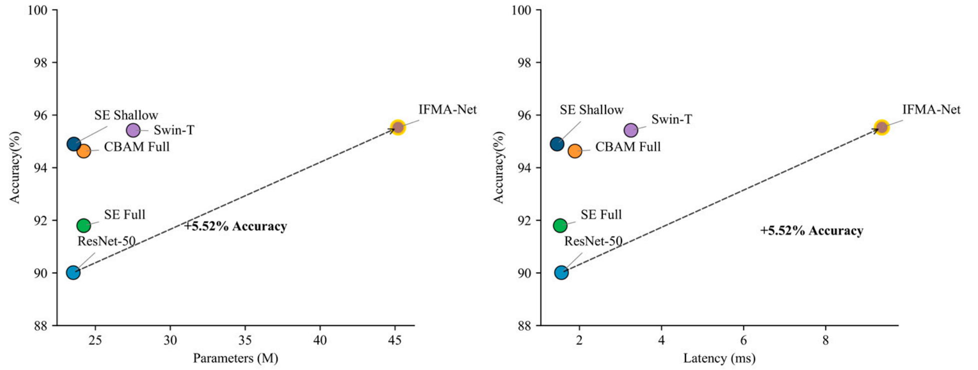

Figure. S1 Accuracy–complexity comparison. Left: accuracy vs. parameters (M); right: accuracy vs. inference latency (ms). The dashed line marks the 5.52-percentage-point accuracy gap between ResNet-50 and IFMA-Net. Latency was measured on an NVIDIA RTX 4090 (batch size = 1, FP32), averaged over three runs.

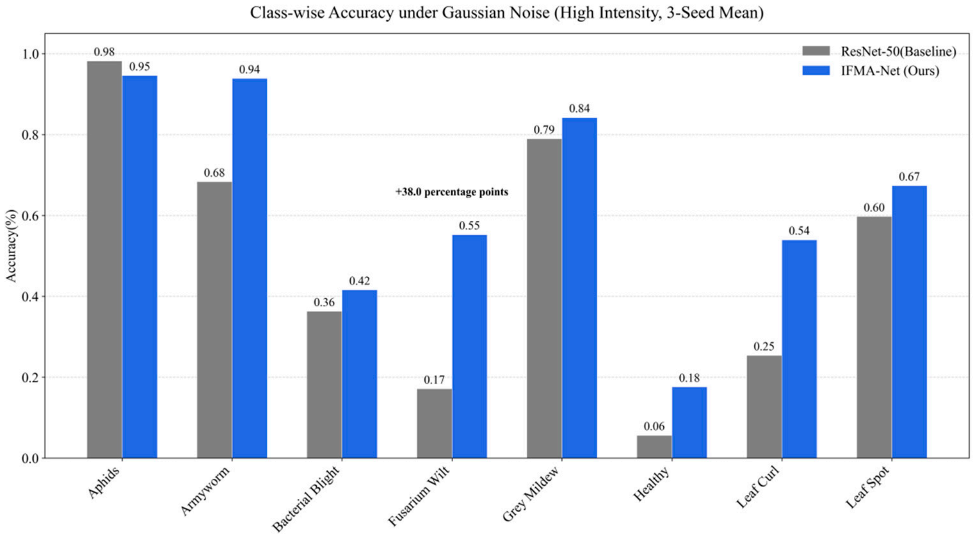

Figure. S2 Class-wise accuracy of ResNet-50 and IFMA-Net under L3 Gaussian noise. Bars show means over three runs using random seeds 42, 123, and 456.

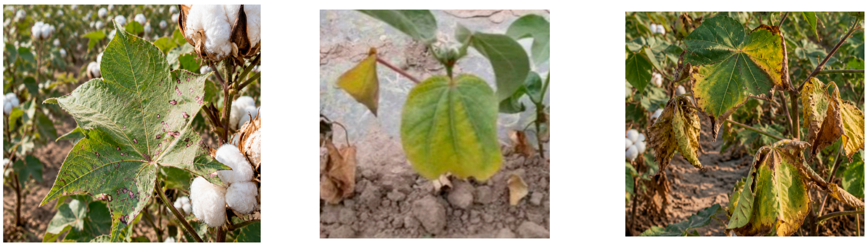

Bacterial Blight Fusarium Wilt Fusarium Wilt

Figure. S3 Representative field symptom examples of cotton Bacterial Blight and Fusarium Wilt. Images are for symptom illustration only.
